# The clinical and economic value of enhanced influenza vaccines for the elderly in Argentina

**DOI:** 10.1016/j.jvacx.2024.100587

**Published:** 2024-11-16

**Authors:** Analía Urueña, Paula Micone, Joaquín Mould-Quevedo, Carolina Saenz, Micaela Delgado, José Luis Montes, Norberto Giglio

**Affiliations:** a Centro de Estudios para la Prevención y Control de Enfermedades Transmisibles (CEPyCET). Universidad ISalud, Buenos Aires, Argentina; b Hospital Carlos G Durand, Buenos Aires, Argentina; c CSL-Seqirus USA, NJ, USA; d CSL-Seqirus Buenos Aires, Argentina; e Hospital de Niños Ricardo Gutiérrez, Buenos Aires, Argentina

**Keywords:** Elderly immunization, Cost-effectiveness analysis, Enhanced influenza vaccines, Argentina

## Abstract

**Background:**

Enhanced influenza vaccines are the best option for the elderly. In 2021, Argentina introduced the MF59-adjuvanted inactivated influenza vaccine (aIIV) for individuals aged 65 years. and above, in the national immunization program. High dose inactivated influenza vaccine (HD-IIV) is also currently registered. This study evaluates the clinical and economic outcomes of these noted enhanced influenza vaccines for the elderly in Argentina.

**Methods:**

Using a static decision-tree model and adopting the payer's perspective during an average influenza season, the analysis incorporated influenza epidemiological data from pre-pandemic Argentinian seasons (2014–2019), strain distribution, vaccination uptake, influenza-related costs and Quality-Adjusted Life-Years (QALYs) gained. Results include two relative vaccine effectiveness (rVE) scenarios from two published meta-analyses, due to reported rVE variability, although without statistical significance expected between enhanced vaccines. Vaccination acquisition costs were obtained from aIIV manufacturer, while HD-IIV costs were estimated using local (Argentinian private sector) and international public sector data (Europe). This assessment considered one GDP per-capita (US$13,696) as a cost-effectiveness threshold and included multiple sensitivity analysis.

**Results:**

With an expected lower vaccine cost and non-significant higher rVE for aIIV vs HD-IIV (3.2 %), aIIV generated 41.4 QALYs gained and US$8.7 M savings to the Argentinean public health system. In this scenario aIIV resulted as a dominant strategy over HD-IIV. On a second scenario, where HD-IIV has a non-significant higher rVE compared to aIIV (15.9 % and 13.9 % for HD-IIV and aIIV, respectively, both vs standard-dose IIV), HD-IIV would only result cost-effective compared to aIIV if its public price is up to 25 % the incremental cost in relation to the standard-dose IIV acquisition price.

**Conclusions:**

In Argentina, the use of enhanced influenza vaccines in the elderly can increase vaccine effectiveness, reduce mortality and disease-related costs. Based on comparable effectiveness, the economic advantage of aIIV over HD-IIV confirms the current vaccination strategy employing aIIV in Argentina.

## Highlights

• In 2021, Argentina introduced the MF59-adjuvanted inactivated influenza vaccine (aIIV) for individuals aged 65 and above in the national immunization program. High dose inactivated influenza vaccine (HD-IIV) is also registered. This study evaluates the clinical and economic outcomes of these enhanced influenza vaccines for the elderly in Argentina.
• The MF59-adjuvanted inactivated influenza vaccine (aIIV), with a lower cost and slightly higher relative vaccine effectiveness (rVE) of 3.2 %, resulted as a dominant strategy over HD-IIV.
• On a second scenario, were HD-IIV has a non-significant higher rVE compared to aIIV (15.9 % vs 13.9 %, vs standard-dose IIV), HD-IIV would only result cost-effective compared to aIIV if its public price is below 50 % incremental cost in relation to the standard-dose IIV acquisition price.
• Enhanced influenza vaccines for the elderly in Argentina improve vaccine effectiveness, reduce mortality, and lower disease-related costs.
• The economic advantages of aIIV over HD-IIV supports Argentina's strategy of using aIIV in the national immunization program.

## 1 Introduction

Seasonal influenza is one of the respiratory viral infections with the highest burden of disease, manifesting in both healthy people and those with comorbidities. The *World Health Organization* estimates that there are around a billion cases of seasonal influenza annually, including 3–5 million cases of severe illness, and that it causes 290,000 to 650,000 respiratory deaths annually. [1]

A J-shaped incidence curve is described for influenza, with a higher incidence rate in both extremes of life compared with the average. However, adults over 65 are by far at greatest risk of influenza-related complications and death. [2] The US Center For Disease Control and Prevention (CDC) reported a cumulative influenza hospitalization rate in 65+ yo of 178 and 215/100,000 population in seasons 2022–2023 and 2023–2024, respectively, which are 2.8 times higher than the overall cumulative incidence of 62 and 79/100,000 population for the same seasons. [3] In addition, in recent years the CDC estimated that between 70 % and 85 % of seasonal flu-related deaths have occurred in people 65 years and older. [4] Also, local data has shown in different seasons that the highest flu death rates are reported in people aged 65+. [5,6]

At present, vaccination remains the most effective intervention to prevent seasonal influenza disease. [1] Vaccination and influenza treatment reduce disease burden by lowering morbidity, mortality, and direct and indirect cost in terms of healthcare resource utilization and productivity lost. [7] However, the effectiveness of traditional influenza inactivated vaccines has not always proven to be as high as needed, especially among the elderly. [8] In this age group, immunosenescence, which refers to the age-related decline in immune system function, may play an important role. [9,10] Thus, the elderly, tend to achieve lower protection from standard inactivated influenza vaccines than other age groups, especially against H3N2, the main subtype associated with peaks in rates of pneumonia and influenza deaths. [11,12]

Enhanced vaccines emerge to mitigate the effects of age-related immunosenescence by providing higher immunogenicity and increased relative vaccine efficacy/effectiveness (rVE) compared with standard vaccines. The adjuvanted trivalent/quadrivalent inactivated influenza vaccines (aIIV), that contain the adjuvant MF59, an oil-in-water emulsion of squalene oil; the high-dose trivalent/quadrivalent inactivated influenza vaccines (HD-IIV), that contain four-times more hemagglutinin antigen than standard-dose vaccines; and the recombinant quadrivalent influenza vaccines (QIVr), that apart from a non-egg, non-cell technology uses a higher antigen content; all of them have demonstrated to increase the magnitude of the immune response and to have a higher relative vaccine efficacy or effectiveness than the standard inactivated influenza vaccine. [[13], [14], [15], [16], [17]]

Vaccination against seasonal influenza has been recommended and free of charge in Argentina since 2011 for high risk groups including children between 6 months to 2 years, adults >65 years, healthcare workers, pregnant women, and subjects with comorbid conditions and chronic diseases between 2 and 64 years. [18] Since then, inactivated influenza vaccines recommended by the World Health Organization (WHO) have been used. The transition from non-adjuvanted trivalent influenza vaccine (TIV) to a trivalent aIIV for the elderly population in Argentina was found to be cost-effective, according to an analytical model developed in 2020. [19] Later that year, the Argentine National Immunization Technical Advisory Group (NITAG), based on epidemiological data, vaccine effectiveness evidence, local cost-effectiveness analyses, and enhanced vaccines availability, recommended that older individuals receive the adjuvanted trivalent influenza vaccine. [20] The Argentine Ministry of Health accepted this recommendation and trivalent aIIV is available in the public healthcare subsector for individuals over 65 years old since 2021. [21]

Currently, another enhanced vaccine recommended for the elderly, the high dose inactivated influenza vaccine (HD-IIV), is also registered in our country but only available in the private sector. The recombinant influenza vaccine is not registered in Argentina. The purpose of this study was to assess the clinical and economic outcomes of adjuvanted and high dose influenza vaccines in people over 65 years old in Argentina from the payer's perspective during an average influenza season.

## 2 Methods

### 2.1 Model and rational Structure

A static decision-tree model was developed with TreeAge Pro 2020 R1.2 software (TreeAge Software LLC., Williamstown, MA, USA).

**Fig. 1:**
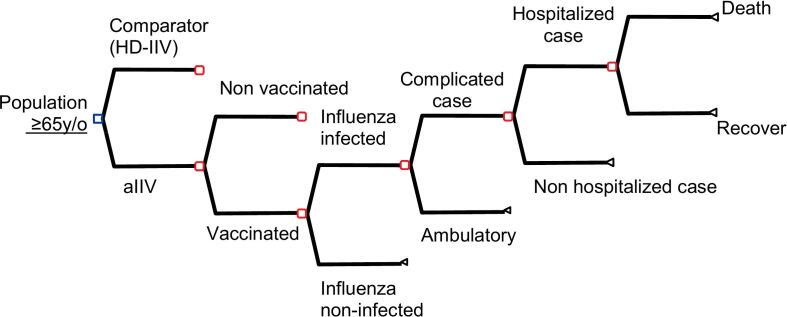
Decision tree model type. aIIV: adjuvanted inactivated influenza vaccine; HD-IIV: high dose inactivated influenza vaccine.

It included a population-based cohort of adults 65 years and older at risk of influenza infection and complications. Main outcomes predicted for each vaccination strategy included the number of influenza cases; general practitioner (GP) visits; complicated ambulatory cases; hospitalizations; deaths; cost of vaccination; direct medical costs; and costs per quality adjusted life years (QALYs) gained. (Fig. 1).

### 2.2 Main inputs

According to the last Census (2022), there are 5,103,968 people 65 years of age and above living in Argentina. [22]

Inputs related to incidence rate, probability of complications and death, influenza A and B distribution, vaccination coverage, direct costs, and utilities (quantitative expression of an individual's preferences for or desirability of a particular state of health relative to perfect health) were obtained from a previous cost-effectiveness analysis led by this same group of authors. [23](Table 1) This analysis included the influenza epidemiological surveillance for the 2014–2019 seasons. The COVID-19 pandemic (2020) and post-pandemic years (2021−2022) were not included as those were considered atypical years.

**Table 1:** Main model inputs.

Variable		Input	Reference
*Population ≥ 65 years*		5,103,968	INDEC. Censo 2022 [22]
*Influenza incidence rate, %*		6.3 %	Urueña et al. Vaccines 2021 [23]
	Complicated cases, %	9.4 %	Urueña et al. Vaccines 2021 [23]
	Hospitalizations, %	36.0 %	Urueña et al. Vaccines 2021 [23]
	Case Fatality Ratio, %	32.0%	Urueña et al. Vaccines 2021 [23]
*Average strain B circulation (2013–2019), %*		18.2 %	Urueña et al. Vaccines 2021 [23]
*Average lineage B match (2013–2019), %*		65.8 %	Urueña et al. Vaccines 2021 [23]
*Vaccine coverage, %*		55.3 %	Urueña et al. Vaccines 2021 [23]
Costs (US$) *			
	*Ambulatory case*	$ 91.0	Updated from Urueña et al. Vaccines 2021 [23]
	*Hospitalized case*	$ 1560.0	Updated from Urueña et al. Vaccines 2021 [23]
	*aIIV vaccine (per dose)*	$ 6.9	Seqirus Argentina 2023
	*QIV vaccine (per dose)*	$ 6.0	PAHO revolving fund, 2023 [25]
	*HD-IIV vaccine (per dose)*	$ 10.0	*Author's estimation based on average value of international vaccine incremental cost relative to QIV (40 %–238 %)* [[26], [27], [28], [29]]

aIIV: adjuvanted inactivated influenza vaccine (trivalent); HD-IIV: high dose inactivated influenza vaccine; SD-QIV: standard dose quadrivalent influenza vaccine. *Costs are expressed as 2023 US dollars.

Costs considered in the model included the management of influenza disease and influenza-related complications. Direct medical costs from previous publication were updated to June 2023 under the consumer's price index application and considering an exchange rate of 1USD = ARS 251.5. [24] People over 65 years old can be retired in Argentina by law. However, a proportion of people in this age group do not effectively retire at this age and continue working. As there is no official information regarding the proportion of the 65+ year-old working population, indirect costs related to opportunity loss were not included for this study, and only the payer's perspective was considered. aIIV acquisition cost was sourced directly from the manufacturer (CSL Seqirus). As there is no official price of HD-IIV for the public sector, it was calculated by averaging the possible incremental costs of this vaccine over the Panamerican Health Organization (PAHO's) revolving fund price for the standard-dose quadrivalent influenza vaccine (SD-QIV). For this estimation we considered information from both local private sector sources (40 % incremental price) and international public sector data (156–238 % incremental price). [[25], [26], [27], [28], [29]] (Table 1).

### 2.3 Relative vaccine effectiveness

Relative vaccine effectiveness of aIIV and HD-IIV compared to the standard-dose inactivated influenza vaccine (SD-IIV) was obtained from the literature. As there are many types of studies, over many seasons, and assessing different outcomes, for the purpose of this analysis we selected three main publications. One randomized clinical trial conducted by Diaz Granados and colleagues assessed the vaccine efficacy of trivalent HD-IIV compared to the SD-IIV against influenza-like illness (ILI) in adults 65 years of age or older during the 2011–2012 and the 2012–2013 northern-hemisphere influenza seasons. [15] The authors reported a relative efficacy of 24.2 %; 95 % confidence interval [CI], 9.7 to 36.5. [15]

A meta-analysis led by Coleman included information from 21 studies that compared the effectiveness of trivalent aIIV to standard or high-dose egg-based influenza vaccines for adults 65 years or older. [30] All studies were conducted in North America or Europe during the 2006/07–2019/20 influenza seasons. In cohort design studies, the effectiveness of trivalent aIIV was significantly higher relative to standard-dose TIV and QIV with pooled estimates of 13.9 % (95 % CI: 4.2, 23.5 %) and 13.7 % (95 % CI: 3.1, 24.2 %), respectively, for any influenza-related medical encounter (Outpatient visits, Emergency Department, or hospitalization). The pooled estimate of the relative VE of trivalent aIIV compared with trivalent HD-IIV included the null and had a relatively narrow confidence interval around it (3.2 %, 95 % CI: −2.5-8.9 %), indicating comparable ability in averting influenza-related medical encounters.

Another meta-analysis published by Lee [31] and collaborators examined the rVE of trivalent HD-IIV compared to SD-IIV against probable or laboratory-confirmed ILI over 6 influenza seasons, including two randomized studies and three observational studies. [31] All studies were performed in North America and were published between 2013 and 2020. Across all seasons, HD-IIV was more effective than SD-IIV in preventing probable or laboratory-confirmed ILI, with a pooled rVE of 15.9 % (95 % CI: 4.1 – 26.3 %). In addition, the authors analyzed fifteen studies that examined the rVE of HD-IIV compared to SD-IIV against hospital admissions and reported that across all studied seasons, HD-IIV was more effective than SD-IIV at preventing hospital admissions due to influenza illness (rVE = 11.7 %; 95 % CI: 7.0–16.1 %).

We decided to include two relative vaccine effectiveness (rVE) scenarios due to the reported variability in these published meta-analyses, although there is no statistical significance expected in vaccine effectiveness beteween enhanced vaccines. (Table 2)

• Scenario 1 considered a 24.2 % relative vaccine efficacy of HD-IIV over the SD-IIV as reported by Diaz Granados and col. [30]. and it included a 3.2 % rVE of aIIV over HD-IIV as described in Coleman's meta-analysis [30]
• Scenario 2 considered 13.9 % and 15.9 % incremental effectiveness of aIIV and HD-IIV, respectively, over the SD-IIV as reported in each meta-analysis.

**Table 2:** Absolute and relative vaccine efficacy/ effectiveness inputs.

Variable		Input	Reference
Vaccine efficacy against influenza A, %			
	Trivalent and quadrivalent	58 % (95 %CI 34;73)	Clements et al. [33]
Vaccine efficacy against influenza B, %			
	Trivalent/ Quadrivalent, match	68 % (95 %CI 15; 99)	Clements et al. [33]
Relative vaccine effectiveness Scenario 1			
	HD-IIV vs SD-IIV, %	24,2 % (95 %CI 9,7;36,5)	Díaz Granados et al. [15]
	aIIV vs HD-IIV, %	3.2 % (95 %CI -2,5;8,9)	Coleman et al. [30]
Relative vaccine effectiveness Scenario 2			
	HD-IIV vs SD-IIV, %	15.9 % (95 %CI 4,2;23,5)	Lee et al. [31]
	aIIV vs SD-IIV, %	13.9 % (95 %CI 4,1;23,6)	Coleman et al. [30]

HD-IIV: high dose inactivated influenza vaccine; SD-IIV: standard dose inactivated influenza vaccine; aIIV: adjuvanted inactivated influenza vaccine.

Theoretical cost-effectiveness threshold was assumed as one gross domestic product per capita (1GDPpc ≈ USD 13,650, year 2022, the last year informed by the World Bank at the time of the analysis). [32]

### 2.4 Uncertainty

Based on the assumptions raised, a deterministic sensitivity analysis (DSA) was carried out in addition to a Monte Carlo simulation with the aim to mitigate the uncertainty regarding the input data to the model. Variables included in the DSA were ranged using the 95 % confidence intervals whenever available, or ± 20 % of the mean if not. The probabilistic sensitivity analyses were performed over 10,000 simulations.

## 3 Results

**Fig. 2:**
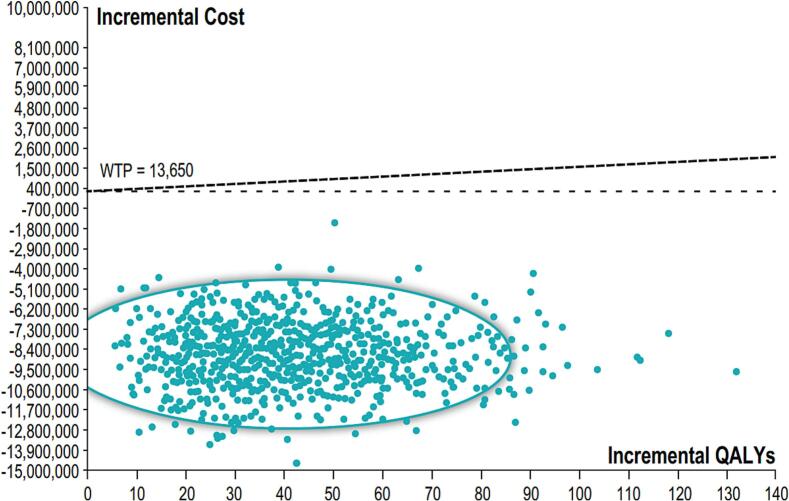
Probabilistic sensitivity analysis. Montecarlo Simulation. Incremental cost-effectiveness aIIV versus HD-IIV. Scenario 1. WTP: willingness to pay; QALYs: Quality adjusted life years.

In the first scenario, with an expected lower vaccine cost and non-significant higher rVE for aIIV (3.2 %), aIIV would result in 41 QALYs gained, it would prevent 800 influenza-related medical encounters (IRME) including 48 complicated ambulatory cases, 27 hospitalizations and 9 deaths annually. It would also generate savings to the Argentinean public health system in US$ 8.7 M. In this scenario aIIV resulted as a dominant strategy over HD-IIV (Table 3) with a 100 % likelihood of aIIV simulations falling below the willingness-to-pay threshold of one GDP per capita. (Fig. 2).

**Table 3:** Main health and economic outcomes. Scenario 1.

		Non vaccinated	HD-IIV	aIIV	Difference aIIV vs HD-IIV
Total events		139, 710	44,432	43,632	−800
Ambulatory cases					
	Non-complicated	126,605	40,264	39,539	−725
	Complicated	8387	2667	2619	−48
Hospitalizations		4717	1500	1473	−27
Deaths		1509	480	471	−9
QALYs gained			24,438,476	24,438,517	41
Net costs			54,116,404	45,338,773	−8,777,631
Incremental Cost-Effectiveness Ratio					Dominant

HD-IIV: high dose inactivated influenza vaccine; aIIV: adjuvanted inactivated influenza vaccine; QALYs: Quality Adjusted Life Years.

**Fig. 3:**
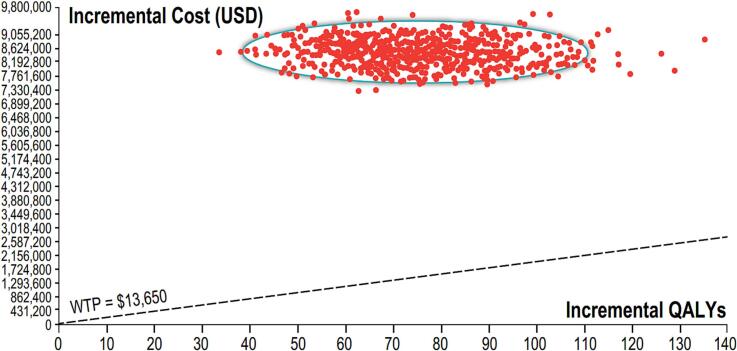
Probabilistic sensitivity analysis. Montecarlo Simulation. Incremental cost-effectiveness HD-IIV versus aIIV. Scenario 2. WTP: willingness to pay; QALYs: Quality adjusted life years.

**Fig. 4:**
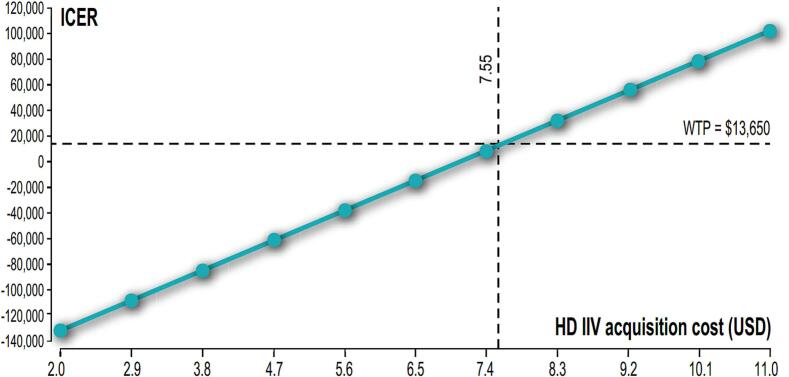
One-way sensitivity analyses, HD-IIV vaccine price and willingness to pay (WTP) threshold (1 GDPpc = USD 13,650). Scenario 2. ICER: Incremental cost-effectiveness ratio; WTP: willingness to pay; HD-IIV: high dose inactivated influenza vaccine.

On a second scenario, where HD-IIV has a non-significant higher rVE compared to aIIV, HD-IIV would prevent 2068 events, including 124 complicated ambulatory cases, 69 hospitalizations and 23 deaths annually, resulting in 107 QALYs gained. However, net costs (the difference between vaccination costs and direct medical costs averted) would result in more than US$ 8.3 M and the incremental cost-effectiveness ratio (ICER) would be US$ 78,245/QALY gained. (Table 4) In this scenario, PSA showed 100 % likelihood of HD-IIV simulations above the willingness-to-pay threshold of one GDP per capita. (Fig. 3) In a one-way sensitivity analysis, and considering one GDPpc (US$13,650) as a willingness to pay, the strategy would only result cost-effective compared to aIIV if the incremental cost of HD-IIV for the public sector does not exceed 25 % over the price of SD-IIV. (Fig. 4).

**Table 4:** Main health and economic outcomes. Scenario 2.

		Non vaccinated	HD-IIV	aIIV	Difference HD-IIV vs aIIV
Total events		139,710	55,081	53,013	−2068
Ambulatory cases					
	Non-complicated	126,605	48,041	49,914	−1873
	Complicated	8387	3182	3306	−124
Hospitalizations		4717	1790	1859	−69
Deaths		1509	572	595	−23
QALYs			24,438,031	24,437,924	107
Net costs			55,322,968	46,948,649	8,374,319
Incremental Cost-Effectiveness Ratio					$78,245

HD-IIV: high dose inactivated influenza vaccine; aIIV: adjuvanted inactivated influenza vaccine; QALYs: Quality Adjusted Life Years.

**Fig. 5:**
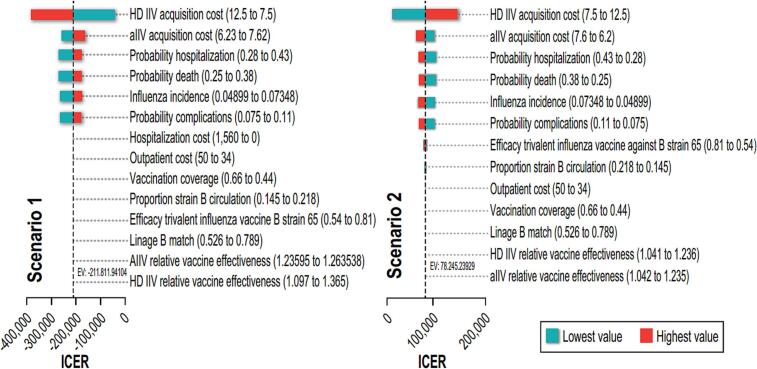
Deterministic sensitivity analyses. Tornado diagram. Scenario 1: aIIV versus HD-IIV; Scenario 2: HD-IIV versus aIIV. HD-IIV: high dose inactivated influenza vaccine; SD-IIV: standard dose inactivated influenza vaccine; aIIV: adjuvanted inactivated influenza vaccine.

In both scenarios vaccine acquisition costs were the only critical determinants of the ICER due to the expected comparable effectiveness between these two vaccines. Probability of hospitalization, complication, or death, as well as influenza incidence, appear as other variables that may influence ICER value, although with much less impact than vaccine price. (Fig. 5).

## 4 Discussion

Given the high burden of disease in older adults it is of high importance to offer them enhanced vaccines that have proven to be more immunogenic and effective than the SD-IIV along multiple seasons and studies. [[13], [14], [15], [16], [17]] Our country implemented enhanced influenza immunization for the elderly with aIIV in 2021, which is offered free of charge for the entire population 65+ year old through the public healthcare subsystem. Another enhanced influenza vaccine, the HD-IIV, is also registered in Argentina, but only available in the private healthcare sector, for those who have health insurance or for those who chose to pay for it. In this study, we explored and compared the health benefits and costs of these two available vaccines from the perspective of the public payer.

This analysis considered two scenarios, one where aIIV exhibited a slightly higher rVE compared to HD-IIV, and the other scenario where HD-IIV demonstrated a higher rVE. In both cases, the 95 % confidence intervals overlapped, indicating that the observed differences are not statistically significant.

Our results showed that when aIIV had a significantly lower acquisition price and similar or slightly higher rVE than the comparator, the strategy turns dominant, and would generate health benefits and net cost savings. On the other hand, when differences in rVE favor HD-IIV but still at a higher cost, the ICER results well above the hypothetical WTP threshold. In this scenario, the strategy would only result in cost-effectiveness when the incremental cost of HD-IIV does not exceed 50 % over the price of SD-IIV. In fact, sensitivity analysis showed that in any of these two scenarios, the main driver of the model is the vaccine's acquisition price.

These findings are in line with those from recent reviews on real world evidence (RWE) in cost-effectiveness analysis of enhanced influenza vaccine in older adults, which showed that adjuvanted and high-dose enhanced vaccines resulted cost-effective compared with standard vaccines, and that differences in relative vaccine effectiveness estimates and acquisition price drove differences in cost-effectiveness results between enhanced vaccines. [34,35]

Our results contrast with other cost-effectiveness analyses that found HD-IIV cost-effective or even dominant compared with aIIV. These studies prioritized that the relative vaccine effectiveness value entered the model are limited only to randomized clinical trials (RCTs) source. As there is no RCT that compares efficacy of aIIV versus SD-IIV, the rVE for this vaccine was set to a very low value or zero in some cases. [[36], [37], [38]]

RCTs have always been considered the best evidence for decision-making, especially in relation to the efficacy of a given pharmacological intervention, based mainly on the minimization of possible confounders by randomly distributing the groups destined for the new intervention or control. However, these studies have considerable limitations. They are very expensive. Although they are characterized by significant internal validity, RTCs sometimes lack external validity, because it is not possible to extrapolate their results to other scenarios or populations, they are performed in short periods of time, they have a limited sample that does not allow the observation of infrequent adverse effects, etc. [39] In the case of influenza vaccination, RCTs may take years to plan, implement, and analyze, and may not produce broadly generalizable findings across influenza seasons and patient populations. RWE, on the other hand, can overcome these issues. They comprise information produced from data routinely collected on a patient's health status and/or delivery of health care from various sources other than traditional clinical trials. [40] Thus, they provide timely and expanding datasets to monitor and evaluate vaccine effectiveness, which is important given the dynamics of a continuously changing influenza virus. RWE research has received attention in the recent decade and is playing an increasing role in health care decisions as shown by the recent COVID-19 pandemic. [41] However, objections to observational studies include the potential for bias from unrecognized factors along with the belief that sometimes these studies overestimate treatment effects. [39] The use of meta-analysis, in this case, can help to increase the number of individuals assessed, include multiple influenza seasons and different populations. In this sense, combining primary studies with varying sample sizes and patient populations can increase the generalization of the results of individual studies. [41]

Thus, in the absence of defining head-to-head clinical trials among enhanced influenza vaccines, for this analysis we chose to include the relative incremental effectiveness between improved vaccines versus the standard influenza vaccine reported by two main meta-analyses. These meta-analyses included both randomized clinical trials and real-world evidence such as cohort, case-control, and test-negative design studies. However, one limitation of these selected meta-analyses is that influenza-related outcomes were not the same. While the study of Coleman et al. compared vaccine effectiveness of aIIV relative to SD-IIV against IRME (outpatient, emergency department visits, and hospitalizations) as a combined primary outcome, the study of Lee and col. analyzed the rVE of HD-IIV compared to SD-IIV against ILI and hospitalizations separately. Another meta-analysis led by Domnich and de Waure that compared effectiveness between aIIV and HD-IIV concluded that, at the moment, both vaccines appear to have similar effectiveness in preventing seasonal influenza in the elderly. [42]

Regulatory entities, National immunization technical advisory groups, and decision makers consider many aspects when making recommendations, such as public health priorities, vaccine safety, effectiveness and cost, the findings of cost-effectiveness analyses, and logistical aspects, guaranteed provision, etc. This may explain why the guidance on vaccine selection varies among countries with multiple and comparable enhanced vaccine options available. The Advisory Committee on Immunization Practices (ACIP), as well as the United Kingdom JCVI, preferentially recommends either aQIV, HD-QIV or QIVr for older adults aged ≥65 years, with no preference expressed for any of these. [43,44] The Australian Technical Advisory Group on Immunization (ATAGI) and the National Advisory Committee on Immunization in Canada recommend for adults aged ≥65 years, both the adjuvanted and high-dose influenza vaccines over standard influenza vaccine, with no preference for use between either of them. [45,46] HD-IIV has a preferential recommendation in Germany (Standing Committee on Vaccination STIKO). [47] Recently, the Health Information and Quality Authority (HIQA) in Ireland, conducted a health technology assessment and concluded that aIIV for the elderly would reduce the burden of influenza and represent the best use of resources in Ireland. [48] In Latin America, Argentina is the only country that included an enhanced influenza vaccine in its regular calendar for the elderly and opted for the aIIV.

Our study has some limitations. We chose not to consider the societal perspective due to uncertainty about the employment rate among the target population. We presumed a majority were retired, thus anticipated that including indirect costs, such as productivity losses, would not substantially alter the outcomes. However, this overlooks potential productivity impacts on other adults responsible for caring for the elderly individual, though estimating such data proves even more challenging.

As there was no reference price for the cost of the HD_IIV vaccine in the Argentinian public sector, it was estimated based on the comparison of its price in Argentina, and other European countries, with the reference value of its standard dose version in those same countries. This estimate may not be accurate. However, the acquisition cost of USD10 used in the base case, that represents a 45 % more than the value of aIIV, is lower than the costs used in other pharmacoeconomic models in both absolute and relative terms.

Finally, we did not specify, in this analysis, whether the inactivated influenza vaccines were trivalent or quadrivalent. Data on the available trivalent aIIV and HD-IIV were assumed to be relevant to quadrivalent aIIV and HD-IIV, respectively, considering that trivalent and quadrivalent formulations of both vaccines are manufactured using the same process and have overlapping compositions. What is more, in the absence of Yamagata lineage circulation since March 2020 following the COVID-19 pandemic, the WHO influenza vaccine composition advisory committee stated in September 2023 that the inclusion of a B/Yamagata antigen as a component of influenza vaccines is no longer warranted, and every effort should be made to exclude it as soon as possible. [49] However, in case Yamagata lineage reemerges in the future, both vaccines have the quadrivalent version and could be available if needed.

On the other hand, as a major strength, our study mainly used official data sources and reviewed local bibliography for population, epidemiological, coverage and cost inputs, which many developing countries lack.

To conclude, the use of enhanced influenza vaccines in the Argentinian elderly population can increase vaccine effectiveness, reduce influenza-related medical encounters and disease-related costs. Based on comparable vaccine effectiveness, significant economic advantages favor the MF59-adjuvanted inactivated influenza vaccine strategy due its expected lower acquisition costs. Head-to-head RCTs or large multi-season observational studies with similar outcomes are warranted to assess significant effectiveness differences between these two enhanced vaccines.

## Funding

This work was funded by CSL Seqirus. CSL Seqirus was involved in the design of the economic model, analysis, and interpretation of results as well as the writing of the manuscript.

## CRediT authorship contribution statement

**Analía Urueña:** Conceptualization, Data curation, Formal analysis, Investigation, Methodology, Project administration, Supervision, Validation, Writing – original draft, Writing – review & editing. **Paula Micone:** Conceptualization, Data curation, Formal analysis, Investigation, Methodology, Software, Validation, Writing – original draft, Writing – review & editing. **Joaquín Mould-Quevedo:** Conceptualization, Methodology, Validation, Writing – original draft, Writing – review & editing. **Carolina Saenz:** Conceptualization, Methodology, Validation, Writing – original draft, Writing – review & editing. **Micaela Delgado:** Conceptualization, Methodology, Validation, Writing – original draft, Writing – review & editing. **José Luis Montes:** Conceptualization, Methodology, Validation, Writing – original draft, Writing – review & editing. **Norberto Giglio:** Conceptualization, Data curation, Formal analysis, Investigation, Methodology, Validation, Writing – original draft, Writing – review & editing.

## Declaration of competing interest

The authors declare the following financial interests/personal relationships which may be considered as potential competing interests:Analia Uruena reports financial support was provided by Seqirus Srl. Analia Uruena reports a relationship with Seqirus Inc. that includes: speaking and lecture fees. Analia Uruena reports a relationship with Merck Sharp & Dohme Corp that includes: speaking and lecture fees. Analia Uruena reports a relationship with Takeda Pharmaceutical Company Limited that includes: speaking and lecture fees. Analia Uruena reports a relationship with GSK that includes: consulting or advisory. Analia Uruena reports a relationship with Takeda Pharmaceutical Company Limited that includes: consulting or advisory. Analia Uruena reports a relationship with Seqirus Inc. that includes: consulting or advisory. Analia Uruena reports a relationship with Sociedad Argentina de Vacunologia y Epidemiologia that includes: board membership. Joaquin Mould Quevedo reports a relationship with Seqirus Inc. that includes: employment and equity or stocks. Carolina Saenz reports a relationship with Seqirus Inc. that includes: employment. Jose Luis Montes reports a relationship with Seqirus Inc. that includes: employment. Norberto Giglio reports a relationship with Argentine Society of Pediatrics that includes: board membership. Norberto Giglio reports a relationship with Ricardo Gutierrez Children's Hospital that includes: board membership. Norberto Giglio reports a relationship with Global Vaccines Data Network that includes: board membership. Norberto Giglio reports a relationship with GSK that includes: consulting or advisory and speaking and lecture fees. Norberto Giglio reports a relationship with Seqirus Inc. that includes: consulting or advisory and speaking and lecture fees. Norberto Giglio reports a relationship with Takeda Pharmaceutical Company Limited that includes: consulting or advisory and speaking and lecture fees. Norberto Giglio reports a relationship with Sanofi Pasteur Inc. that includes: consulting or advisory and speaking and lecture fees. If there are other authors, they declare that they have no known competing financial interests or personal relationships that could have appeared to influence the work reported in this paper.

## Data Availability

Data will be made available on request.
